# Survival impact of microsatellite instability in stage II gastric cancer patients who received S-1 adjuvant monotherapy after curative resection

**DOI:** 10.1038/s41598-023-37870-y

**Published:** 2023-07-04

**Authors:** Chihiro Sato, Hisato Kawakami, Ryo Tanaka, Hironaga Satake, Kentaro Inoue, Yutaka Kimura, Junya Fujita, Ryohei Kawabata, Yasutaka Chiba, Taroh Satoh, Kazuhiko Nakagawa

**Affiliations:** 1 Department of Medical Oncology, Kindai University Faculty of Medicine, 377-2 Ohno-Higashi, Osaka-Sayama, Japan; 2 Laboratory of Molecular Immunology, Institute for Quantitative Biosciences, The University of Tokyo, Bunkyo, Japan; 3 Department of General and Gastrointestinal Surgery, Osaka Medical and Pharmaceutical University, Takatsuki, Japan; 4 Cancer Center, Kansai Medical University Hospital, Hirakata, Japan; 5 Department of Medical Oncology, Kochi Medical School, Kochi, Japan; 6 Department of Surgery, Kansai Medical University Hospital, Hirakata, Japan; 7 Department of Surgery, Faculty of Medicine, Kindai University, Osaka-Sayama, Japan; 8 Department of Surgery, Kindai University Nara Hospital, Ikoma, Japan; 9 Department of Surgery, Yao Municipal Hospital, Yao, Japan; 10 Department of Surgery, Sakai City Medical Center, Sakai, Japan; 11 Department of Surgery, Osaka Rosai Hospital, Sakai, Japan; 12 Clinical Research Center, Kindai University Hospital, Osaka-Sayama, Japan; 13 Center for Cancer Genomics and Precision Medicine, Osaka University Hospital, Suita, Japan

**Keywords:** Chemotherapy, Gastric cancer, Tumour biomarkers

## Abstract

Adjuvant S-1 monotherapy is the standard of care for stage II gastric cancer (GC) after curative resection in Japan, but its efficacy for microsatellite instability–high (MSI-H) tumors has remained unknown. Among a multi-institutional cohort of patients with stage II GC who underwent R0 resection followed by S-1 adjuvant chemotherapy between February 2008 and December 2018, we assessed MSI status with an MSI-IVD Kit (Falco). MSI status was assessable for 184 (88.5%) of the 208 enrolled patients, with MSI-H being identified in 24 (13.0%) individuals. Although neither relapse-free survival (RFS) (hazard ratio [HR] = 1.00, *p* = 0.997) nor overall survival (OS) (HR = 0.66, *p* = 0.488) differed between MSI-H versus microsatellite-stable (MSS) patients, MSI-H patients showed a nonsignificant but better RFS (HR = 0.34, *p* = 0.064) and OS (HR = 0.22, *p* = 0.057) than did MSS patients after adjustment for background characteristics by propensity score (PS) analysis. Gene expression analysis in the PS-matched cohort suggested that recurrence was associated with the immunosuppressive microenvironment in MSI-H tumors but with expression of cancer/testis antigen genes in MSS tumors. Our data reveal a better adjusted survival for MSI-H versus MSS stage II GC treated with S-1 adjuvant therapy, and they suggest that mechanisms of recurrence differ between MSI-H and MSS tumors.

## Introduction

Gastric cancer (GC) remains one of the most common and deadly cancers worldwide. In Japan, it is the third most common cause of cancer death, with an estimated 43,000 such deaths in 2019. In attempts to improve the survival of GC patients, several combinations of chemotherapeutic agents have been adopted for adjuvant treatment of stage III GC after curative D2 lymph node dissection. On the other hand, for stage II GC, 1-year adjuvant chemotherapy with the oral fluoropyrimidine S-1 has been the standard treatment in Japan based on the ACTS-GC study^1^, which showed 3-year relapse-free survival (RFS) and overall survival (OS) rates of 72.2% and 80.1%, respectively.

DNA mismatches that occur during cell division are largely restricted to microsatellite regions of the genome, which are composed of repetitive sequences of 2 or 3 bases. Defects in the mechanism of mismatch repair (MMR) can affect the number of such repeats in microsatellite regions, with such an abnormality being known as microsatellite instability (MSI). Tumors with a high MSI are designated as MSI-high (MSI-H)^2^, with the MSI-H phenotype usually being due to a deficiency in MMR caused by germline mutations in MMR-related genes including *MLH1*, *MSH2*, *MSH6*, and *PMS2* or, more commonly, by epigenetic silencing of *MLH1* as a result of its hypermethylation. The former mechanism underlying MSI-H status is more prevalent in younger individuals, with the latter being associated with aging^2^. The Cancer Genome Atlas network has classified GC according to four molecular subtypes: Epstein-Barr virus–infected tumors, MSI tumors, genomically stable tumors, and tumors with chromosomal instability^3^. Among these subtypes, MSI tumors have received attention for their potential immune reactivity, given the recent development of immune checkpoint inhibitors (ICIs).

Clinical and molecular profiling of MSI-H tumors has been largely limited to colorectal cancer (CRC)^4,5^. In early-stage CRC, MSI-H is a prognostic indicator for survival and for resistance to fluoropyrimidines, with guidelines now stating that patients with MSI-H stage II disease do not require adjuvant therapy^6^. In the case of GC, the importance of postoperative adjuvant chemotherapy for MSI-H tumors is currently under debate^7,8^. An integrated analysis that included the CLASSIC^9^ and MAGIC^10^ phase 3 trials showing the benefit of peri- or postoperative adjuvant chemotherapy for stage II/III GC suggested that MSI-H patients do not benefit from adjuvant chemotherapy as much as do microsatellite-stable (MSS) patients^11^. Other retrospective studies have also suggested that 5-fluorouracil–based adjuvant chemo(radio)therapy does not provide a disease-free survival benefit in patients with MSI-H GC^12–14^. However, these various studies were performed for stage II/III GC and with various chemotherapeutic regimens. No study has previously evaluated 5-fluorouracil monotherapy for MSI-H stage II GC, with such a study being especially important for patients in Japan, where S-1 monotherapy is the standard of care for adjuvant treatment of stage II GC. Furthermore, the true incidence as well as the characteristics of MSI-H stage II GC have remained unclear.

We have therefore now retrospectively investigated the prevalence and characteristics of MSI-H stage II GC and assessed the efficacy of S-1 adjuvant chemotherapy for such tumors compared with MSS stage II GC.

## Results

### Patients

**Figure 1:**
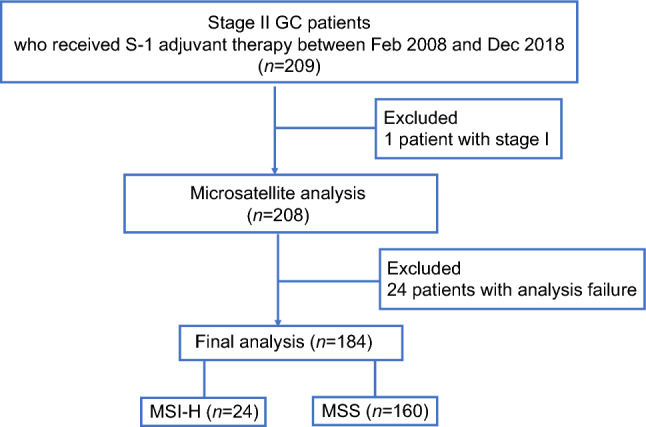
Flow diagram for study participants.

A total of 209 eligible patients was enrolled in the study. To identify MSI-H cases, we screened for MSI status in the 208 tumor samples from the stage II GC patients who received S-1 monotherapy as adjuvant chemotherapy. MSI status was evaluable in 184 samples (88.5%), with MSI-H being detected in 24 cases, yielding a prevalence of MSI-H in the study population of 13.0% (24/184) (Fig. 1). The characteristics of the MSI-H and MSS patients are shown in Table 1. MSI-H patients were older (median age of 70 vs. 67 years, *p* = 0.016) and tended to have a higher T stage but lower N stage compared with MSS patients, with all MSI-H cases being stage IIb (compared with 56.9% for MSS cases, *p* < 0.001). The neutrophil-to-lymphocyte ratio (median of 2.25 vs. 1.69, *p* = 0.086) and serum C-reactive protein concentration (median of 0.12 vs. 0.09 mg/dL, *p* = 0.543) were numerically higher, whereas the serum albumin level (mean of 3.78 vs. 3.96 g/dL, *p* = 0.021) was significantly lower, in the MSI-H patients than in the MSS patients. Reflecting their generally poor background, dose reduction for S-1 was more frequent in MSI-H patients than in MSS patients (25.0% vs. 16.9%, *p* = 0.391), resulting in the duration of S-1 therapy being similar in the two groups (median of 321.0 vs. 327.5 days, respectively). The most common reasons for S-1 discontinuation in MSI-H or MSS patients were adverse events (80% in each group) followed by disease recurrence (10% vs. 9%, respectively).

**Table 1:** Patient characteristics. Data are number (percent) unless indicated otherwise.

Characteristic	Subgroup	MSI-H	MSS	*p* value
		(*n* = 24)	(*n* = 160)	
Sex	Male	17 (70.8)	111 (69.4)	1
	Female	7 (29.2)	49 (30.6)	
Median age (years)		70	67	0.016
Lauren classification	Intestinal	8 (33.3)	48 (30.0)	0.132
	Indeterminate	2 (8.3)	2 (1.2)	
	Diffuse	2 (8.3)	30 (18.8)	
	Mixed	12 (50.0)	80 (50.0)	
Primary tumor location	Upper	2 (8.3)	19 (11.9)	0.721
	Middle	14 (58.3)	100 (62.5)	
	Lower	8 (33.3)	41 (25.6)	
Surgery	Total gastrectomy	5 (20.8)	46 (28.7)	0.543
	Distal gastrectomy	19 (79.2)	113 (70.6)	
	Other	0 (0.0)	1 (0.6)	
T	1	0 (0.0)	19 (11.9)	0.014
	2	3 (12.5)	43 (26.9)	
	3	15 (62.5)	85 (53.1)	
	4a	6 (25.0)	13 (8.1)	
N	0	6 (25.0)	41 (25.6)	0.780
	1	15 (62.5)	82 (51.2)	
	2	3 (12.5)	33 (20.6)	
	3	0 (0.0)	4 (2.5)	
Stage	IIa	0 (0.0)	69 (43.1)	< 0.001
	IIb	24 (100.0)	91 (56.9)	
Performance status	0	18 (75.0)	130 (81.2)	0.580
	1	6 (25.0)	30 (18.8)	
Dose modification	Yes	6 (25.0)	27 (16.9)	0.391
	No	18 (75.0)	133 (83.1)	
Median S-1 duration (days)	321.0	327.5	0.062	
mGPS	0	14 (58.3)	118 (79.2)	0.034
	1	7 (29.2)	26 (17.4)	
	2	3 (12.5)	5 (3.4)	
Median NLR		2.25	1.69	0.086
Mean serum albumin (g/dL)		3.78	3.96	0.021
Median serum CRP (mg/dL)		0.12	0.09	0.543
Recurrence	Yes	3 (12.5)	26 (16.2)	0.772
	No	21 (87.5)	134 (83.8)	
Cancer-specific death		1 (4.2)	19 (11.9)	0.480

*mGPS* modified Glasgow prognostic score, *NLR* neutrophil-to-lymphocyte ratio, *CRP* C-reactive protein (Abbreviations not defined in text).

### Prognosis of MSI-H versus MSS stage II GC

**Figure 2:**
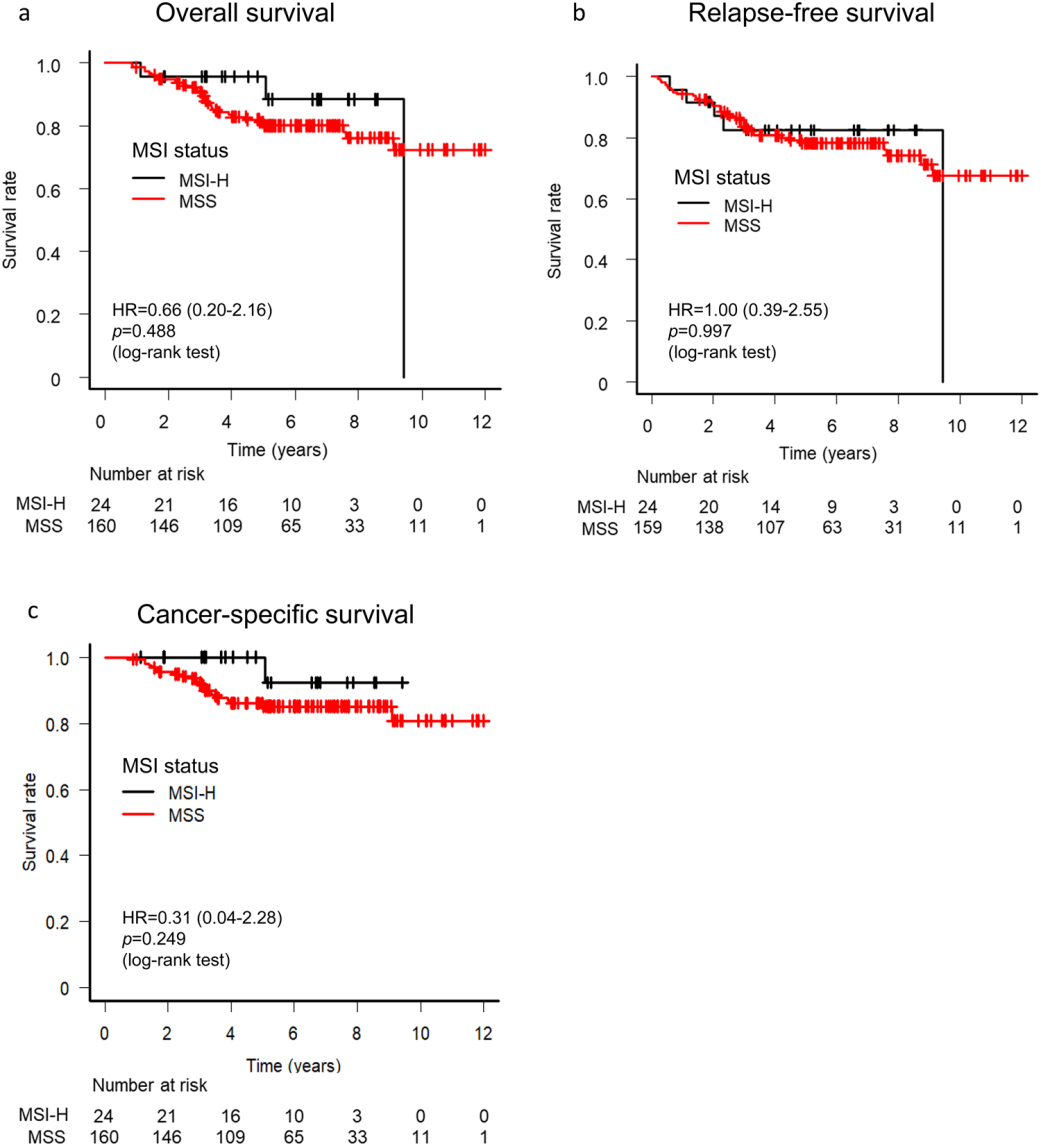
Overall survival (**a**) and relapse-free survival (**b**) for the study subjects according to MSI status.

We assessed the survival of patients with stage II GC who received S-1 monotherapy after curative D2 resection. The 3-year OS and RFS rates for the entire study population were 91.6% (95% confidence interval [CI], 86.4–94.8%) and 83.6% (95% CI, 77.2–88.3%), respectively (Supplementary Figure 1). There was no significant difference in OS (hazard ratio [HR] of 0.66, with a 95% CI of 0.20–2.16; log-rank *p* = 0.488) or in RFS (HR of 1.00, with a 95% CI of 0.39–2.55; log-rank *p* = 0.997) between MSI-H and MSS cases (Fig. 2), with 3-year OS rates of 95.8% and 90.9% and 3-year RFS rates of 82.5% and 83.7% for MSI-H and MSS, respectively.

We further investigated the clinical characteristics of MSI-H versus MSS stage II GC after curative resection (Table 1). There were 3 versus 31 total OS events for MSI-H versus MSS, respectively, with cancer-related death accounting for 1 (33%) versus 19 (61%) of these cases, respectively. The remaining two deaths for the MSI-H cases were due to other causes. Although 26 patients (16.2%) in the MSS group experienced disease recurrence, only 3 patients (12.5%) did so in the MSI-H group. Recurrence sites were varied and included intraperitoneal lymph nodes and liver, or both. Consequently, we analyzed cancer-specific survival (CSS) (Fig. 2c). Although there were no significant differences (hazard ratio [HR] of 0.31 with a 95% CI of 0.04–2.28; log-rank *p* = 0.249) between MSI-H and MSS cases, there was a trend towards better survival in MSI-H patients.

**Figure 3:**
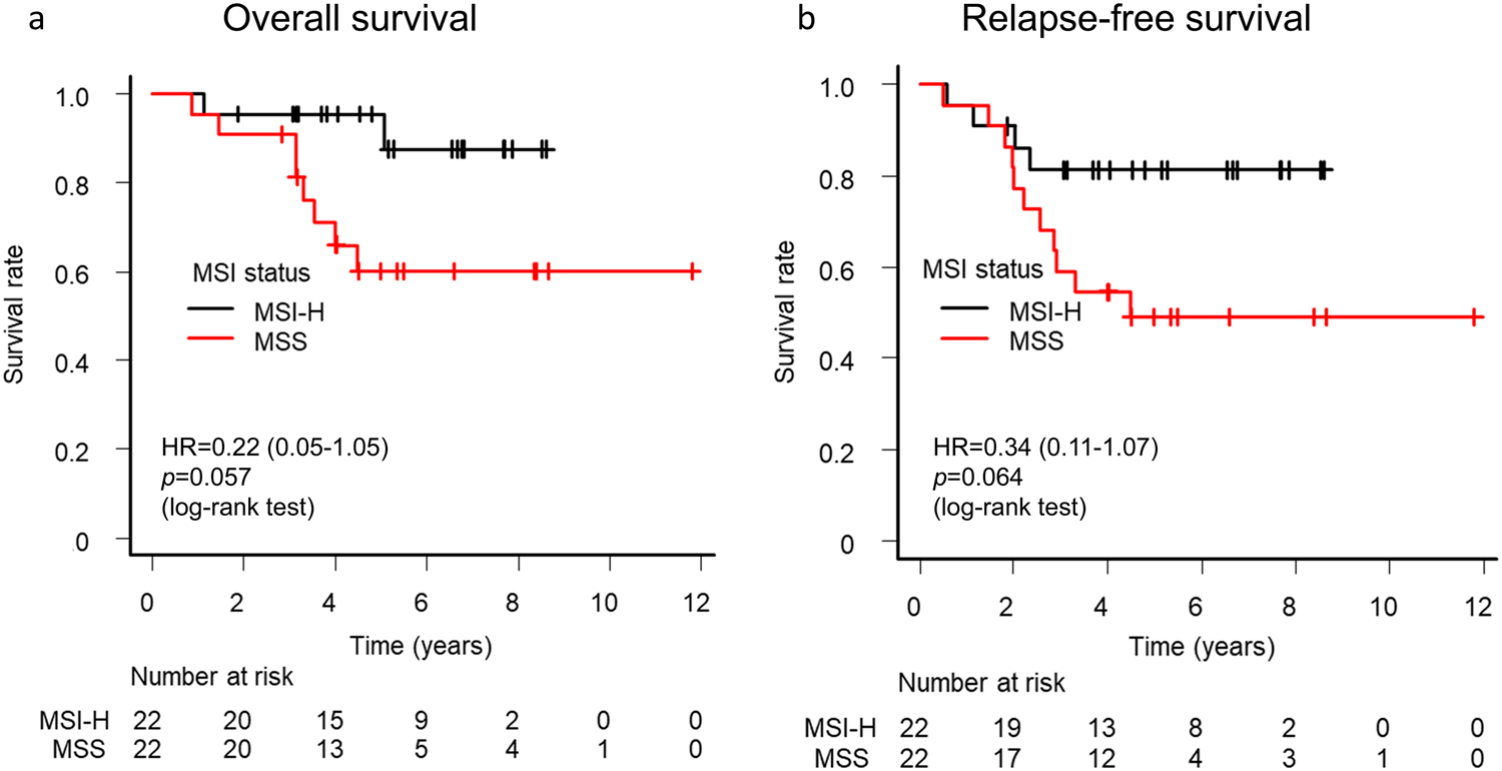
Overall survival (**a**) and relapse-free survival (**b**) according to MSI status with adjustment by propensity score analysis.

Given that there were differences in patient background between the MSI-H and MSS groups, we performed propensity score (PS) analysis to correct for potential imbalances. The PS was calculated with a logistic regression model and with sex, age, T stage, N stage, Lauren classification, and S-1 dose modification as explanatory variables. Weighted characteristics for the MSI-H and MSS groups are shown in Supplementary Table 1. After adjusting for the imbalances, we found a better survival for MSI-H versus MSS cases in terms of 3-year OS rate (95.5% vs. 90.9%; HR, 0.22 [95% CI, 0.05–1.05]; log-rank *p* = 0.057) and 3-year RFS rate (81.3% vs. 59.1%; HR, 0.34 [95% CI, 0.11–1.07]; log-rank *p* = 0.064), although these differences did not achieve statistical significance likely as a result of the small sample size (Fig. 3).

### Differences in gene expression related to recurrence in MSI-H versus MSS stage II GC

**Figure 4:**
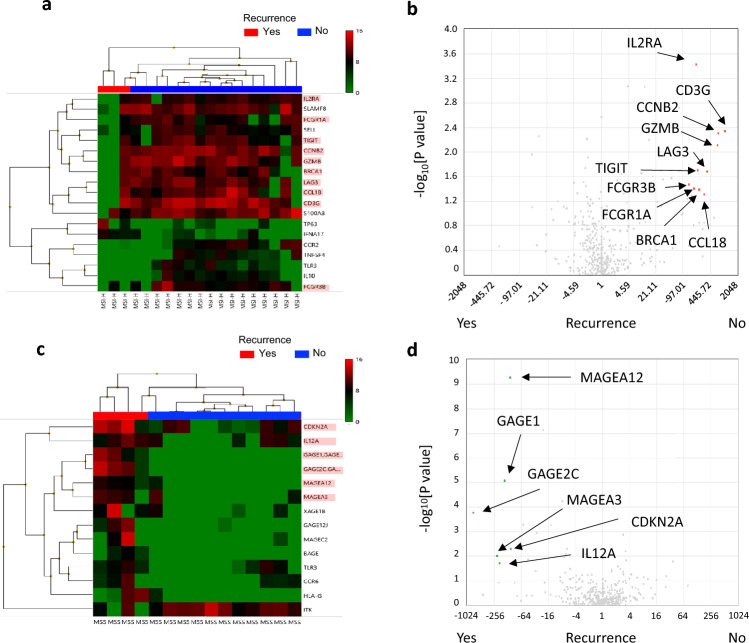
Gene expression in recurrent or nonrecurrent cases according to MSI status in the PS-matched population. Heat maps of immune-related gene expression (**a**, **c**) are shown together with corresponding volcano plots (**b**, **d**) for recurrent or nonrecurrent cases of MSI-H (**a**, **b**) and MSS (**c**, **d**) GC. Each colored square in the heat maps represents the average log_2_[RPM] for expression of the indicated genes in a given patient, with the highest expression indicated in red, median in black, and lowest in green. Genes with a fold change in expression level of more than ± 10 and a *p* value of < 0.05 were extracted (Supplementary Table 2) and are shown in the heat maps, among which those with a fold change of more than ± 100 are highlighted in red in the heat maps and indicated in the volcano plots.

To assess possible differences in recurrence mechanisms between MSI-H and MSS GC, we analyzed gene expression according to recurrence for the PS-matched population. Among tumor samples of the PS-matched cohort, 34 samples (19 MSI-H and 15 MSS tumors), including 7 recurrent cases (3 MSI-H and 4 MSS tumors), were evaluable for such analysis with the Oncomine Immune Response Research Assay. We first compared gene expression between recurrent and nonrecurrent cases, and we found that recurrence was associated with higher expression of cancer/testis antigen (CTA) genes including *MAGEA12*, *GAGE1*, and *GAGE2C* as well as with lower expression of *GZMK* (Supplementary Figure 2 and Supplementary Table 2). We then compared gene expression associated with recurrence according to MSI status. Of note, gene expression patterns differed markedly between MSI-H and MSS tumors. Among MSI-H tumors, nonrecurrent cases showed higher expression of immune response–related genes including *FCGR1A*, *FCGR3B*, *GZMB*, *CD3G*, and *CCL18* as well as of immune checkpoint genes such as *TIGIT* and *LAG3* (Fig. 4a,b and Supplementary Table 2), suggesting that an adverse change in the tumor immune microenvironment may be associated with recurrence. On the other hand, among MSS tumors, recurrence was associated with higher expression of CTA genes (Fig. 4c,d and Supplementary Table 2). These findings thus suggested that the mechanisms underlying recurrence may differ between MSI-H and MSS tumors treated with adjuvant S-1 monotherapy.

## Discussion

As far as we are aware, our retrospective study is the first to examine the impact of MSI status on the survival of stage II GC patients who receive S-1 adjuvant monotherapy after curative resection. A recent large cohort study revealed the prevalence of MSI-H in GC to be 6.74% (130/1929) in a Japanese population, in which most cases were recurrent or metastatic^15^. In the present study, MSI-H was identified in 13% of completely resected stage II GC tumors of Japanese patients, suggesting that the prevalence of MSI-H is higher in early-stage tumors and decreases with stage progression, as has been shown for CRC^4,5^. Consistent with previous findings^16^, we also found that MSI-H GC cases were common in the distal portion of the stomach and were associated with the intestinal subtype of the Lauren classification as well as with a higher T stage rather than N stage, suggestive of the absence of clear racial or ethnic differences in clinicopathologic characteristics of MSI-H GC. In the current study, MSI-H patients were significantly older than MSS patients, suggesting that hypermethylation of MMR-related genes may be a cause of MSI-H in GC and hence that the condition is sporadic. With regard to age, we also found that nutrition and inflammation status appeared to be worse in MSI-H cases than in MSS cases, with such factors usually being associated with a poor survival^17^. Furthermore, S-1 dose reduction was required more frequently at the beginning of the adjuvant treatment for MSI-H cases, resulting in a lower S-1 dose intensity compared with MSS cases. Of interest, however, we found that the survival of MSI-H patients was not inferior to that of MSS patients in the current study. Moreover, survival was markedly better for MSI-H patients than for MSS patients after adjustment for patient background by PS analysis. We also found that cancer-specific death was numerically lower in the MSI-H group than in the MSS group.

Whereas S-1 monotherapy has been the standard of care for stage II GC after curative resection based on the ACTS-GC study in Japan^1^, little has been known of the impact of MSI status on the survival of such treated patients. Our data now suggest that MSI testing may be of benefit for elderly patients with stage II GC, given that MSI-H patients, even those with relatively poor backgrounds, have an excellent prognosis with S-1 postoperative adjuvant chemotherapy. However, it is unclear whether this is due to the inherent good prognosis of MSI-H or to S-1. In this regard, MSI analysis may provide insight into the value of S-1 monotherapy for MSI-H GC in an ongoing study (JCOG1507, BIRDIE study, jRCTs031180255) in which adjuvant S-1 monotherapy is compared with gastric resection alone in vulnerable elderly patients with stage II/III GC.

Our gene expression analysis revealed a potential difference in the mechanisms of recurrence between MSI-H and MSS tumors treated with adjuvant S-1 monotherapy, with recurrence in the former tumors likely being associated with immune suppression in the tumor microenvironment. We found increased CD3G and FCGR1A/3B expression, indicating that immune cells such as T lymphocytes, B lymphocytes, and macrophages were abundant in non-relapsed cases of MSI-H. Furthermore, the expression of GZMB and IL2R, which are the result of the immune response, was also high in MSI-H non-relapsed cases. These results suggest that T cells in tumors from non-relapsed cases of MSI-H were in the early stage of activation and had antitumor activity. Although LAG3 and TIGIT are commonly used as markers of exhausted T cells, these markers are first expressed when T cells are activated by antigen stimulation and are persistently present on the cell surface to gradually exhaust T cells^18^. Considering other gene expressions indicative of an active immune response, elevations in these markers were likely evidence of immune activity. Although MSI-H tumors in general are thought to have a good prognosis at early stages, data suggest that they have a poor prognosis in the absence of T cell infiltration^19^. It remains to be determined whether the S-1 adjuvant treatment impacted positively or negatively on such immune suppression tumors apparent in recurrent MSI-H cases in the present study. Developments in perioperative treatment with ICIs for early MSI-H tumors are under way^20^. Although the efficacy of such ICI-based therapy is generally high for these tumors, it is possible that the clinical benefit is limited to those cases with a poor immune environment, given that MSI-H tumors inherently have a good prognosis^20^. On the other hand, the expression of CTA genes was associated with recurrence in MSS tumors, consistent with previous results showing that the expression of *MAGE* and *GAGE* genes is associated with poor prognosis in GC^21–23^. Given their tumor-specific expression, immunotherapy targeting the proteins encoded by these genes has also been developed (jRCT2080222504). Nonetheless, our data suggest that MSI-H and MSS gastric tumors constitute distinct populations and therefore require the development of different treatment strategies.

Our study has several limitations. First, in this study, we performed PSM with MSI status as the objective variable and sex, age, T stage, N stage, Lauren classification and S-1 dose modification as the explanatory variables, which are clinically considered to have a particular impact on prognosis. Mainly due to sample size, there were limitations in adjusting for background variation with one-to-one matching, especially in making the SMD sufficiently small for factors other than the six explanatory factors listed above. However, the differences were generally smaller than before matching (although not necessarily as small as the SMD value), indicating that it is worthwhile to compare survival rates after adjusting for patient background. Second, the design of the study was retrospective and single arm, lacking data for patients treated with surgery alone. This lack is due to the fact that only samples obtained after S-1 adjuvant therapy became the standard (in 2008) were analyzed so as to ensure DNA quality, with the success of MSI analysis indeed being 88.5% in the present study. Furthermore, patients who still did not receive S-1 adjuvant therapy after the ACTS-GC study appeared to have a markedly different background, such as a poor performance status, and we therefore excluded these individuals. Second, we did not include patients with stage III GC. Our aim was to assess the relation between survival and MSI status among patients who received S-1 monotherapy. Given that various adjuvant regimens—such as docetaxel plus S-1^24^, capecitabine plus oxaliplatin^9^, and S-1 plus oxaliplatin^25^—have been administered and that neoadjuvant regimens have recently been evaluated^26,27^ for stage III GC, data for the effect of MSI status on the efficacy of S-1 adjuvant chemotherapy for stage III GC will likely be of little clinical relevance. Moreover, perioperative therapy with ICIs plus cytotoxic agents has been evaluated for stage II/III GC^28,29^. A study of preoperative treatment with ipilimumab plus nivolumab for MSI-H GC including that of stage II has recently demonstrated substantial efficacy^30^ but lacked a chemotherapy cohort as a control. These factors underscore the importance of our data as a reference for future potential adoption of such approaches in clinical practice in Japan.

In summary, we found the prevalence of MSI-H to be 13% in Japanese patients with stage II GC who received S-1 adjuvant chemotherapy. MSI-H status was associated with stage IIb disease and a poor general condition, including a significantly older age and lower serum albumin concentration. Despite such indicators of a poorer prognosis compared with MSS stage II GC, patients with MSI-H GC showed a similar or—after adjustment for such background factors by PS analysis—better survival outcome. Given the potential difference in mechanisms of recurrence between MSI-H and MSS tumors, further study of such mechanisms is warranted, especially with the ongoing evaluation of ICIs for perioperative therapy in GC.

## Methods

### Patients

Patients included in this study were those who with stage II GC who received S-1 monotherapy as adjuvant chemotherapy after curative D2 resection at five affiliated institutions in Osaka between February 2008 and December 2018. Data regarding clinicopathologic features and treatment history were extracted. We also collected information on general status and laboratory data obtained immediately before adjuvant S-1 administration. Eligible patients were diagnosed with pathological stage II GC according to the 15th edition of the Japanese classification of GC and received S-1 monotherapy as adjuvant chemotherapy on a 1-year schedule after the standard curative D2 lymph node dissection in accordance with corresponding guidelines. Patients who had stage III GC or who received neoadjuvant chemotherapy were excluded. Histological classification and primary site were also determined according to the guidelines. This study was approved by the Institutional Review Board (IRB) of Kindai University Faculty of Medicine (approval number 30–130) in accordance with the Declaration of Helsinki. Patients provided written informed consent, where applicable, or such consent was waived by the IRB of Kindai University Faculty of Medicine, given that the study was retrospective in nature and made use only of deidentified data and excess archival tissue.

### Microsatellite analysis

Tumor tissue corresponding to primary GC was collected. The samples were submitted to SRL (CLIA-certified, CAP-accredited central laboratory) for MSI testing with an MSI-IVD Kit (Falco). The procedure included extraction of DNA from normal and tumor components of paraffin-embedded gastrectomy tissue, and MSI status was evaluated by analysis of five microsatellite markers: BAT25, BAT26, NR21, NR24, and MONO27. MSI status was determined as MSI-H if two or more markers were positive. All results were anonymized by SRL, and the aggregated results for MSI status were analyzed in this study.

### Gene expression analysis

Sample RNA was extracted from formalin-fixed and paraffin-embedded sections with the use of a MagMAX FFPE DNA/RNA Ultra Kit (Applied Biosystems) and KingFisher Flex Purification System (Thermo Fisher Scientific), and it was quantified with the use of a Qubit instrument and Qubit RNA High Sensitivity (HS) Kit (Invitrogen). Sequencing libraries were constructed with an Ion Torrent Oncomine Immune Response Research Assay Kit (Applied Biosystems). In brief, RNA (10 ng) was subjected to reverse transcription and the resulting cDNA was amplified with primers for the Oncomine assay. Amplicons were digested, ligated to barcode adaptors, and purified with the use of the AMPure XP reagent (Applied Biosystems). Libraries were quantified by real-time polymerase chain reaction analysis and diluted to a concentration of 50 pM before templating on the Ion Torrent Ion Chef instrument and sequencing with the Ion Torrent Ion GeneStudio S5 system and Ion Torrent Ion 540 Kit-Chef. Data were analyzed with the Torrent Server Immune Response RNA Plugin to provide housekeeping and target gene expression levels in absolute read counts, and target gene expression was normalized by housekeeping gene expression as reads per million (RPM) and log_2_[RPM]. Sample correlation and target gene expression (RPM) clustering heat maps and principal component analysis plots of the top 50 genes with the highest variance were also provided.

### Statistical analysis

All statistical analysis was performed with EZR (Saitama Medical Center, Jichi Medical University, Saitama, Japan), which is a graphical user interface for R (The R Foundation for Statistical Computing, Vienna, Austria)^31^. Differences in categorical variables were assessed with the chi-square test or Fisher’s exact test, and those in continuous variables were evaluated with the independent *t* test. A *p* value of < 0.05 was considered statistically significant. The primary statistical outcome was RFS measured in months from the date of surgery to recurrence, death, or final follow-up. RFS curves were estimated with the Kaplan–Meier method and were compared with the log-rank test. OS was also measured from surgical treatment to death from any cause or last follow-up. Patients without documented clinical or radiographic disease recurrence or who were still alive were censored on the date of last follow-up. Follow-up was closed in December 2020. To minimize potential biases from background differences between the MSI-H and MSS groups, the nearest-neighbor matching method using propensity scores was employed. Logistic regression was used for the propensity score calculation from the following variables: sex, age, T stage, N stage, Lauren classification, and S-1 dose modification. A 1:1 matching by propensity scores was performed using a caliper of 0.20. The balance was verified by assessing a *p* value and standardized mean differences (SMDs).

### Ethics approval and consent to participate

The study was performed according to the Declaration of Helsinki and was approved by the Institutional Review Board of Kindai University Faculty of Medicine (approval number 30–130). Patients provided written informed consent, where applicable, or such consent was waived by the Institutional Review Board–approved protocols, given that the study was retrospective in nature and made use only of deidentified data and excess archival tissue.

## Supplementary Information

Supplementary Information 1.

Supplementary Information 2.

Supplementary Information 3.

Supplementary Information 4.

## Data Availability

The data sets generated during and/or analyzed during the study are not publicly available due to restrictions of the research ethics protocol but are available from the corresponding author upon reasonable request.
