# Supplementary material for: Survival impact of microsatellite instability in stage II gastric cancer patients who received S-1 adjuvant monotherapy after curative resection
Source: Sci Rep. 2023 Jul 4;13:10826. doi: 10.1038/s41598-023-37870-y (PMC10319738; doi:10.1038/s41598-023-37870-y)
Supplement: Supplementary file 1 — Supplementary Information 1. [file 41598_2023_37870_MOESM1_ESM.pptx]

## Slide 1
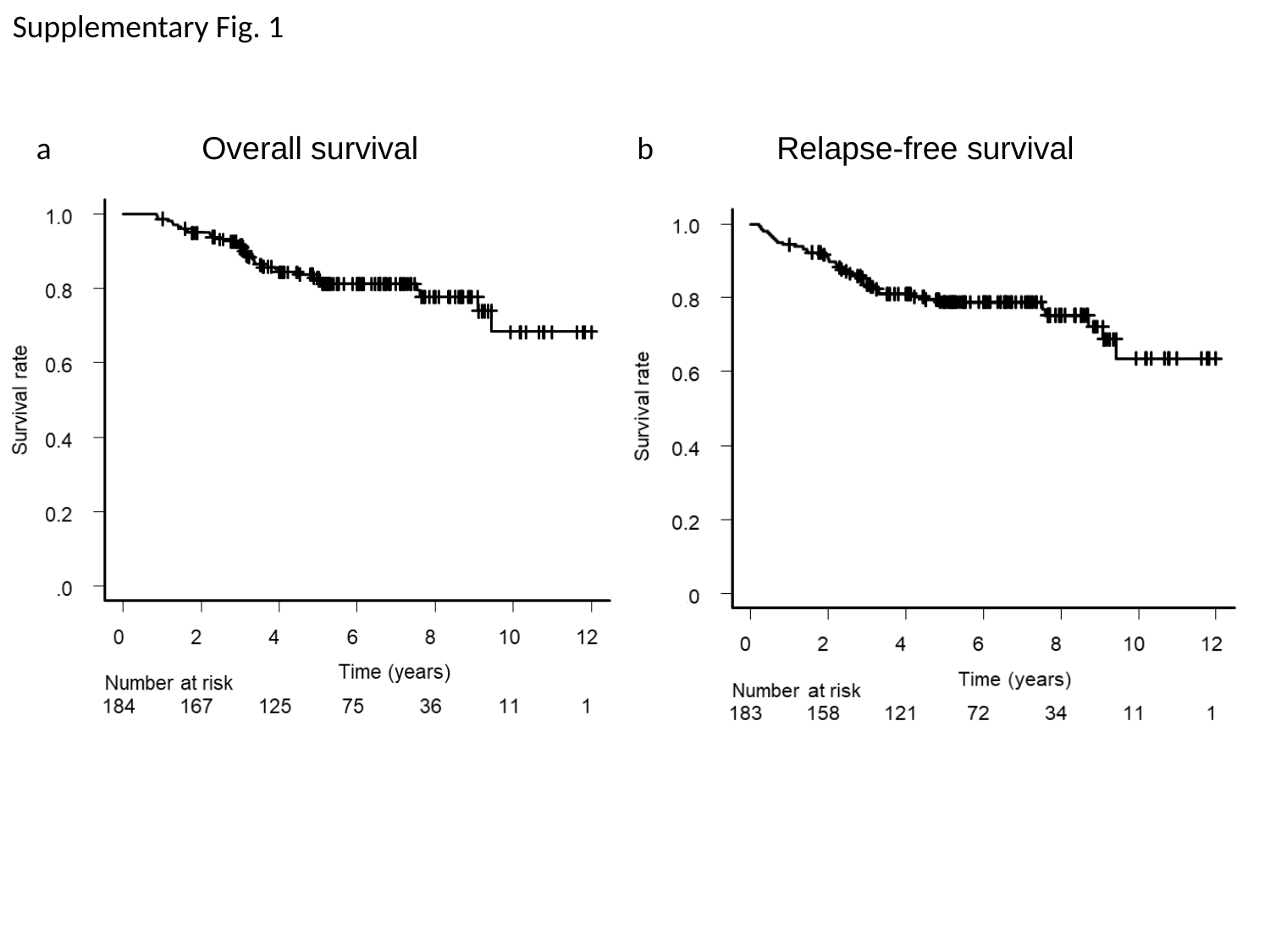

Supplementary Fig. 1
a
b
Relapse-free survival
Overall survival

## Slide 2
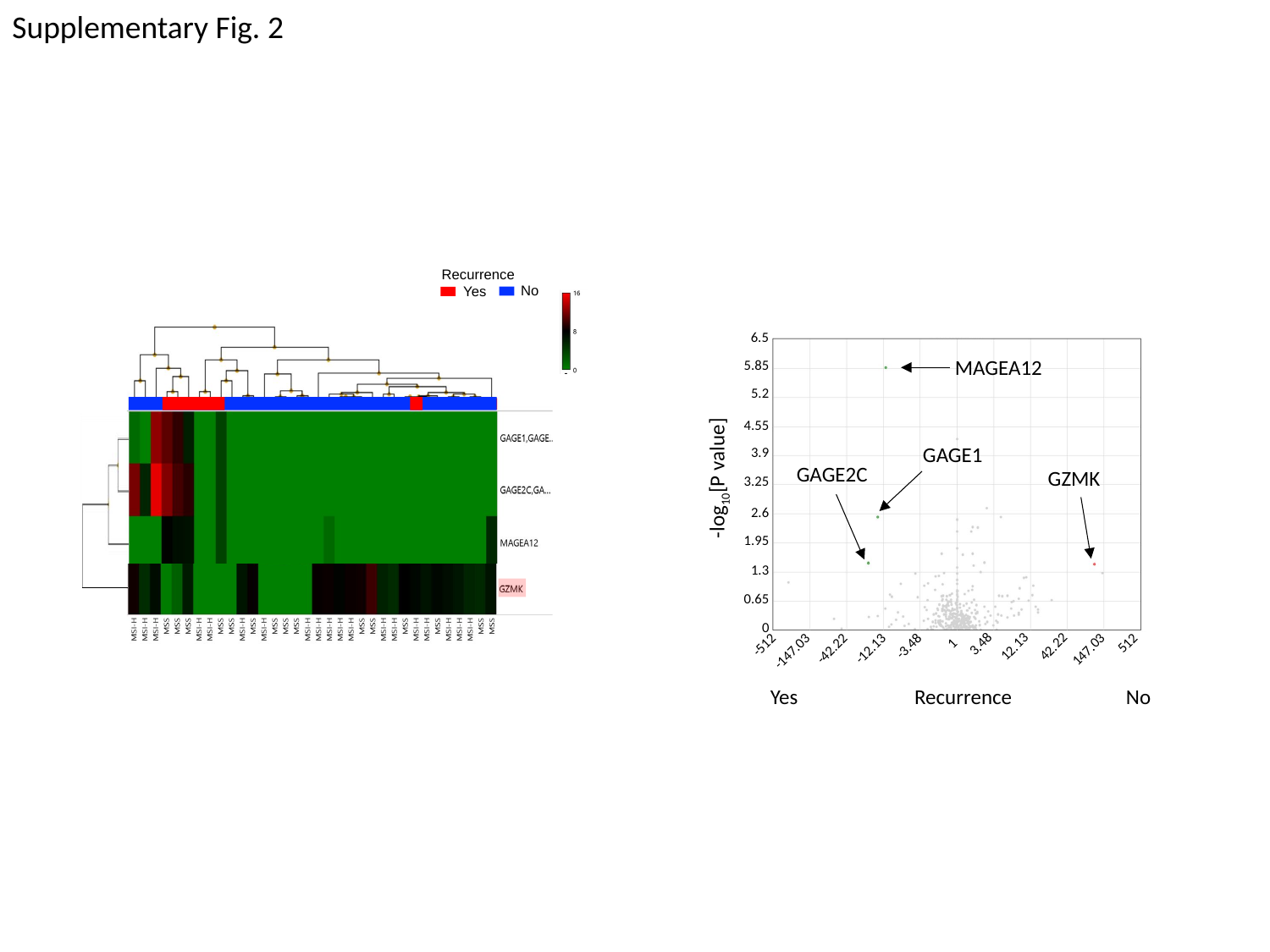

Supplementary Fig. 2
Recurrence
No
Yes
6.5
MAGEA12
5.85
5.2
4.55
GAGE1
3.9
-log10[P value]
GAGE2C
GZMK
3.25
2.6
1.95
1.3
0.65
0
3.48
1
-3.48
-512
-147.03
-42.22
-12.13
12.13
42.22
147.03
512
Yes
Recurrence
No
