# Supplementary material for: Survival impact of microsatellite instability in stage II gastric cancer patients who received S-1 adjuvant monotherapy after curative resection
Source: Sci Rep. 2023 Jul 4;13:10826. doi: 10.1038/s41598-023-37870-y (PMC10319738; doi:10.1038/s41598-023-37870-y)
Supplement: Supplementary file 2 — Supplementary Information 2. [file 41598_2023_37870_MOESM2_ESM.docx]

**Supplementary Table 1.** Patient characteristics after propensity score matching. Data are number (percent) unless indicated otherwise.

|  |  | | **MSI-H** | **MSS** | **p value** | **SMD** |
| --- | --- | --- | --- | --- | --- | --- |
| **Characteristic** | **Subgroup** | | **(n = 22)** | **(n = 22)** |  | **（SMD before matching）** |
| Sex | Male | | 16 (72.7) | 15 (68.2) | 1 | 0.100 |
|  | Female | | 6 (27.3) | 7 (31.8) |  | (0.032) |
| Median age (years) |  | | 70 | 71 | 0.518 | 0.193  (0.559) |
| Lauren classification | Intestinal | | 7 (31.8) | 8 (36.4) | 0.793 | 0.336 |
|  | Indeterminate | | 2 (9.1) | 2 (9.1) |  | (0.445) |
|  | Diffuse | | 2 (9.1) | 4 (18.2) |  |  |
|  | Mixed | | 11 (50.0) | 8 (36.4) |  |  |
| Primary tumor location | Upper | | 2 (9.1) | 3 (13.6) | 0.682 | 0.280 |
|  | Middle | | 14 (63.6) | 11 (50.0) |  | (0.189) |
|  | Lower | | 6 (27.3) | 8 (36.4) |  |  |
| Surgery | Total gastrectomy | | 5 (22.7) | 7 (31.8) | 0.736 | 0.205 |
|  | Distal gastrectomy | | 17 (77.3) | 15 (68.2) |  | (0.219) |
|  | Other | | 0 (0.0) | 0 (0.0) |  |  |
| T | 1 | | 0 (0.0) | 0 (0.0) | 0.692 | 0.285 |
|  | 2 | | 3 (13.6) | 4 (18.2) |  | (0.781) |
|  | 3 | | 15 (68.2) | 12 (54.5) |  |  |
|  | 4a | | 4 (18.2) | 6 (27.3) |  |  |
| N | 0 | | 4 (18.2) | 6 (27.3) | 0.692 | 0.285 |
|  | 1 | | 15 (68.2) | 12 (54.5) |  | (0.340) |
|  | 2 | | 3 (13.6) | 4 (18.2) |  |  |
|  | 3 | | 0 (0.0) | 0 (0.0) |  |  |
| Stage | IIa | | 0 (0.0) | 1 (4.5) | 1.000 | 0.309 |
|  | IIb | | 22 (100.0) | 21 (95.5) |  | (1.213) |
| Performance status | 0 | | 17 (77.3) | 15 (68.2) | 0.736 | 0.205 |
|  | 1 | | 5 (22.7) | 7 (31.8) |  | (0.152) |
| Dose modification | Yes | | 5 (22.7) | 4 (18.2) | 1.000 | 0.113 |
|  | No | | 17 (77.3) | 18 (81.8) |  | (0.201) |
| Median S-1 duration (days) | | | 311 | 328 | 0.213 | 0.340  (0.345) |
| mGPS^{a}^ | | 0 | 12 (54.5) | 18 (90.0) | 0.024 | 0.901 |
|  | | 1 | 7 (31.8) | 2 (10.0) |  | (0.491) |
|  | | 2 | 3 (13.6) | 0 (0.0) |  |  |
| Median NLR | |  | 2.30 | 1.69 | 0.100 | 0.519  (0.257) |
| Mean serum albumin (g/dL) | |  | 3.76 | 3.95 | 0.074 | 0.553  (0.479) |
| Median serum CRP (mg/dL) | |  | 0.16 | 0.12 | 0.480 | 0.413  (0.177) |
| Recurrence (%) | | Yes | 3 (13.6) | 8 (36.4) | 0.162 | 0.544 |
|  | | No | 19 (86.4) | 14 (63.6) |  | (0.107) |

Abbreviations not defined in text: mGPS, modified Glasgow prognostic score; NLR, neutrophil-to-lymphocyte ratio; CRP, C-reactive protein; SMD, standardized mean　difference

^a^mGPS of two patients in the MSS group could not be obtained.
