# Supplementary material for: Survival impact of microsatellite instability in stage II gastric cancer patients who received S-1 adjuvant monotherapy after curative resection
Source: Sci Rep. 2023 Jul 4;13:10826. doi: 10.1038/s41598-023-37870-y (PMC10319738; doi:10.1038/s41598-023-37870-y)
Supplement: Supplementary file 3 — Supplementary Information 3. [file 41598_2023_37870_MOESM3_ESM.docx]

**Supplementary Figure Legends**

**Supplementary Figure 1.** Overall survival (**a**) and relapse-free survival (**b**) for the study population.

**Supplementary Figure 2. Gene expression in recurrent or nonrecurrent cases in the PS-matched population.** A heat map of immune-related gene expression (left) is shown together with a corresponding volcano plot (right) for recurrent or nonrecurrent cases. Each colored block in the heat map represents the average log_2_[RPM] for expression of the indicated genes in a given patient, with the highest expression indicated in red, median in black, and lowest in green. Genes with a fold change in expression level of more than ±10 and a *p* value of <0.05 were extracted (**Supplementary Table 2**) and are shown in the heat map, among which those with a fold change of more than ±100 are highlighted in red in the heat maps and indicated in the volcano plots.
