# Supplementary material for: Survival impact of microsatellite instability in stage II gastric cancer patients who received S-1 adjuvant monotherapy after curative resection
Source: Sci Rep. 2023 Jul 4;13:10826. doi: 10.1038/s41598-023-37870-y (PMC10319738; doi:10.1038/s41598-023-37870-y)
Supplement: Supplementary file 4 — Supplementary Information 4. [file 41598_2023_37870_MOESM4_ESM.docx]

**Supplementary Table 2. Immune-related gene expression in the propensity score–matched population.**

Fold change of more than ±10 and *p* value of <0.05 for recurrent cases (Yes, *n* = 7) versus nonrecurrent cases (No, *n* = 27)

| **Gene ID** | **No, average log_2_[RPM]** | **Yes, average log_2_[RPM]** | **Fold change** | ***p* value** | **FDR** | **Gene symbol** | **Description** |
| --- | --- | --- | --- | --- | --- | --- | --- |
| MAGEA12_30130 | 0 | 3.49 | ­–11.21 | 1.34E–06 | 0.0005 | MAGEA12 | melanoma_antigen_family_A_12 |
| GAGE1_144260 | 0 | 3.87 | –14.6 | 0.003 | 0.2238 | GAGE1, GAGE12I, GAGE12F | G_antigen_1 |
| GAGE2C_104217 | 0 | 4.33 | –20.11 | 0.0317 | 0.7754 | GAGE2C, GAGE2A, GAGE2E | G_antigen_2C |
| GZMK_178286 | 6.8 | 0.05 | 107.19 | 0.0331 | 0.7754 | GZMK | granzyme_K_granzyme_3_tryptase_II |

Fold change of more than ±10 and *p* value of <0.05 for MSI-H tumors from recurrent cases (Yes, *n* = 3) versus those from nonrecurrent cases (No, *n* = 16).

| IL2RA_240338 | 7.66 | 0 | 201.72 | 0.0004 | 0.1156 | IL2RA | interleukin_2_receptor_alpha |
| --- | --- | --- | --- | --- | --- | --- | --- |
| S100A8_239334 | 9.23 | 5.75 | 11.22 | 0.0009 | 0.1156 | S100A8 | S100_calcium_binding_protein_A8 |
| SLAMF8_9071016 | 8.71 | 2.4 | 78.98 | 0.0019 | 0.1874 | SLAMF8 | SLAM_family_member_8 |
| CD3G_616717 | 9.96 | 0 | 996.77 | 0.0046 | 0.2495 | CD3G | CD3g_molecule_gamma_CD3-TCR_complex |
| CCNB2_9861095 | 9.43 | 0 | 691.43 | 0.0049 | 0.2495 | CCNB2 | cyclin_B2 |
| TP63_143246 | 0 | 5.01 | –32.19 | 0.0055 | 0.2495 | TP63 | tumor_protein_p63 |
| SELL_139245 | 7.07 | 3.23 | 14.3 | 0.0063 | 0.2495 | SELL | selectin_L |
| GZMB_581688 | 9.38 | 0 | 668.31 | 0.0077 | 0.2784 | GZMB | granzyme_B_granzyme_2_cytotoxic_T-lymphocyte-associated_serine_esterase_1 |
| IFNA17_691788 | 0 | 5.57 | –47.55 | 0.0109 | 0.3603 | IFNA17 | interferon_alpha_17 |
| TIGIT_383491 | 7.81 | 0 | 224.33 | 0.0198 | 0.4991 | TIGIT | T_cell_immunoreceptor_with_Ig_and_ITIM_domains |
| LAG3_13111419 | 8.56 | 0 | 377.2 | 0.0207 | 0.4991 | LAG3 | lymphocyte-activation_gene_3 |
| TLR3_24932595 | 4.16 | 0 | 17.82 | 0.0251 | 0.5171 | TLR3 | toll-like_receptor_3 |
| CCR2_385483 | 5.64 | 0 | 49.99 | 0.0265 | 0.5171 | CCR2 | chemokine_C-C_motif_receptor_2 |
| IL10_491598 | 4.95 | 0 | 31.02 | 0.0276 | 0.5171 | IL10 | interleukin_10 |
| FCGR3B_620722 | 7.06 | 0 | 133.62 | 0.0335 | 0.5249 | FCGR3B | Fc_fragment_of_IgG_low_affinity_IIIb_receptor_CD16b |
| TNFSF4_347439 | 4.02 | 0 | 16.22 | 0.0347 | 0.5249 | TNFSF4 | tumor_necrosis_factor_ligand_superfamily_member_4 |
| FCGR1A_547652 | 7.5 | 0 | 180.65 | 0.0394 | 0.5393 | FCGR1A | Fc_fragment_of_IgG_high_affinity_Ia_receptor_CD64 |
| BRCA1_42344338 | 7.92 | 0 | 241.68 | 0.0407 | 0.5393 | BRCA1 | breast_cancer_1_early_onset |
| CCL18_198296 | 8.31 | 0 | 318.15 | 0.048 | 0.5622 | CCL18 | chemokine_C-C_motif_ligand_18_pulmonary_and_activation-regulated |

Fold change of more than ±10 and *p* value of <0.05 for MSS tumors from recurrent cases (Yes, *n* = 4) compared with those from nonrecurrent cases (No, *n* = 11)

| MAGEA12_30130 | 0 | 6.82 | 112.77 | 5.31E–10 | 2.11E–07 | MAGEA12 | melanoma_antigen_family_A_12 |
| --- | --- | --- | --- | --- | --- | --- | --- |
| BAGE_153290 | 0 | 4.37 | 20.73 | 7.00E–08 | 1.39E–05 | BAGE | B_melanoma_antigen_1_precursor |
| GAGE1_144260 | 0 | 7.23 | 149.98 | 8.34E–06 | 0.0011 | GAGE1, GAGE12I, GAGE12F | G_antigen_1 |
| GAGE2C_104217 | 0 | 9.52 | 732.81 | 0.0002 | 0.0131 | GAGE2C, GAGE2A, GAGE2E | G_antigen_2C |
| GAGE12J_144260 | 0 | 5.84 | 57.47 | 0.0005 | 0.0297 | GAGE12J | G_antigen_12J |
| MAGEC2_249358 | 0 | 3.82 | 14.08 | 0.0005 | 0.0297 | MAGEC2 | melanoma_antigen_family_C_2 |
| HLA-G_483585 | 0 | 4.87 | 29.21 | 0.0011 | 0.0558 | HLA-G | major_histocompatibility_complex_class_I_G |
| XAGE1B_469547 | 0 | 6.11 | 68.95 | 0.0015 | 0.0616 | XAGE1B | X_antigen_family_member_1B |
| CDKN2A_696829 | 4.23 | 11.01 | 109.68 | 0.0052 | 0.1717 | CDKN2A | cyclin-dependent_kinase_inhibitor_2A_isoform_p12 |
| MAGEA3_20121 | 0 | 7.79 | 221.61 | 0.01 | 0.2207 | MAGEA3 | melanoma_antigen_family_A_3 |
| TLR3_24932595 | 0 | 5.5 | 45.28 | 0.0135 | 0.244 | TLR3 | toll-like_receptor_3 |
| ITK_563669 | 8.6 | 3.42 | –36.06 | 0.0167 | 0.2762 | ITK | IL2-inducible_T-cell_kinase |
| IL12A_739842 | 0 | 7.6 | 193.68 | 0.0196 | 0.3116 | IL12A | interleukin_12A_natural_killer_cell_stimulatory_factor_1_cytotoxic_lymphocyte_maturation_factor_1_p35 |
| CCR6_271363 | 0 | 4.94 | 30.7 | 0.041 | 0.5392 | CCR6 | C-C_chemokine_receptor_type_6 |

FDR, false discovery rate.
